# The National Pension Fund

**Published:** 1887-10-22

**Authors:** 


### THE NATIONAL PENSION FUND.

By our Special Reporters.

On Wednesday evening, the 12th inst., The Hospitals, Association held the first General Meeting of its fifth session, in the rooms of the Society of Arts, John Street, Adelphi. Arrangements had been made for the meeting to take place in the Library, but before the hour fixed for the opening of the proceedings the audience had grown so large that it was deemed advisable to invite the company to adjourn to the large hall. Even this capacious chamber proved none too large for the very numerous attendance Ladies, as might be imagined, largely predominated. Amongst them were the representatives of no less than thirty-five hospitals, including the London, St. Thomas's, Charing Cross, King's College, Westminster, Middlesex, St. Mary's, Paddington; Seamen's, Greenwich; Royal Free; Victoria Hospital for Children, Chelsea; Royal Hospital for Children and Women; Chelsea Hospital for Women; the Women's Hospital, Soho; Alexandra Hospital for Children; Evelina Hospital for Children; Paddington Green Children's Hospital; Sydenham Hospital for Children; Queen Charlotte's General Lying-in Hospital; London Fever Hospital; Eastern Fever Hospital; St. John's Hospital for Skin Diseases; Poplar Hospital for Accidents; City of London Hospital for Diseases of the Chest; Westminster Ophthalmic Hospital; the Royal London Ophthalmic Hospital; Brompton Cancer Hospital; the Lock Hospital: Blackheath Cottage Hospital; Chesham Cottage Hospital, and the Boston Cottage Hospital; twelve infirmaries, including those of Chelsea, Kensington, Marylebone, Notting Hill, Paddington, St. George's-in-the-East, St. Pancras, Shoreditch, Whitechapel, Wandsworth, Bethnal Green, Hampstead, Leicester, and Chelmsford and Essex; and thirteen nursing and kindred institutions.

The chair was taken by Sir Andrew Clark, Bart., M.D., F.R.S., who was supported by Lord Sandhurst, Mr. George King, of the Atlas Assurance Company; Col. Montefiore, Dr. G. W. Potter. Lady Clark and the Hon. Edith Boscawen were also present.

Sir Andrew Clark, who was warmly received, said: It is within my knowledge that this subject has engaged a considerable portion of Mr. Burdett's time for many years. I dare say the paper may be found to be a little difficult to follow, but I beg that, in return for the care and time he has devoted to it, you will give your close attention to its reading.

At the conclusion of the paper (*vide* The Hospital, p. 40) Mr. Burdett said:—I now come to the most difficult part of the subject which has caused delay in the scheme. The difficulty lies, as all these matters do lie, in the question of cost of participation.

**The Cost of Participation.** I have felt that it would be ridiculous to propose a scheme which should be altogether outside the means of those who desire to participate, and yet my friend Mr. King will tell you that there are no such stubborn things as figures, except possibly facts. We have to secure a sound financial basis, and there are absolute figures from which we dare not depart. It is a question of hard fact. How much will so many pounds sterling produce at a given age? Well, I thought I might probably get some help if I consulted you and those who are not here, but who belong to your body. I therefore communicated with all the nursing institutions throughout the country, and I have here a statement which gives the pensions expected and the premiums offered. How much assistance I got out fo these suggestions you will understand when I take a sample or two. Here is a nurse who does not give her age, but who wants a pension of £40, and is prepared to pay £1 a year for a certain time. I admire the enterprise of the lady in question, but it is out of my power to gratify her wish. Then we go on and find that some people's ideas are very different from others. For instance, our friend who wants £40 a year, and offers a pound, is eclipsed by a lady from a London hospital who desires £100 per annum, and is willing to contribute ten shillings a year to get it. Now, difference of view is brought out in another way. In the cottage hospitals they are supposed to have fine air and plenty of time for mature reflection, but these conditions do not always produce identical results, because here are two ladies who both desire pensions of £40, but one lady considers she will give one guinea, and the other, under the same surroundings, with the same wages, thinks, on the whole, she could give three guineas. These results will show you how absolutely impracticable, how widely different are the views which people hold with reference to what is possible and what is necessary.

**The Circumstances of the Nurses.** I have been very much struck, in considering the question of cost with the circumstances of the nurses (I am going all through this paper to dwell chiefly upon the nurses, although the Fund is intended for hospital officials generally. Well, these women, as you know, usually reside in palatial buildings, and I take it that their friends, in consequence, think that their incomes are palatial also. They make all sorts of claims upon them for relief and support. I do sympathise most deeply with the affection and love and regard which many nurses have for their friends, but after all there is a limit, and that limit should not be passed by any individual even when dealing with his own flesh and blood. I do say, and say most earnestly, that I hope this Fund, if it is ever established, will be a protection to nurses and other workers in institutions, because it will place a greater limit upon the available funds which they have and can give away to others. It will, I trust, make them just to themselves. I take it that the object of the Fund is not immediately and at once to make every nurse happy by leading her to think —"I am giving a pound to the Fund, and therefore whatever happens I am provided for for life." The main object is rather to make a substantial and safe commencement to a great movement which will one day result in giving adequate provision in the case of permanent sickness and in old age. Its ultimate result will be to give all an adequate pension. It will furnish all with a sense of their responsibilities, by inducing them to make provision for sickness and old age by paying a sum which shall entitle them to £10 or £15 a year. I believe this Fund will direct the attention of committees and managers of institutions, and the outside public, to the non-provision now existing for workers among the sick. If so, I make bold to say that the managers of the Fund will soon have placed at their disposal sufficient funds to enable them to supplement what individuals can give. You must not think that the statement which I have made this evening is a disappointing statement. It is really a very hopeful one. Nothing that is worth having is ever got without work and lapse of time, the twin sisters of progressive growth. Now, I want to secure a steady progressive growth for this Fund, so that everyone joining will feel that she has absolute security for her money, and that she will get what she pays for when she wants it.

**Two Points of Importance.** There are two other things which should be brought out. First, the advantage of a small number at the outset. I do not suppose we shall get the whole 7,500 or 10,000 nurses and officials to join at once. As a matter of fact and of business, it is not altogether desirable that they should all so join, because we may be able to give better results when we have had experience and seen the class of people we have to deal with, so far as their collective health is concerned. The tables upon which we have calculated are not based upon nurses resident in public institutions, but upon societies such as the Odd Fellows, and therefore your circumstances may be slightly different. The fact that you are well fed, clothed, and housed may result in the sickness among you proving much less than among the class upon whom these tables are based. As far as human prescience can guide us, there is nothing wrong. The financial basis is sound, and what you give you will get back when you want it. It is proposed to make provisions of four kinds for members. First, sickness, (*a*) temporary, (*b*) permanent. Second, pensions, (*a*) with the return of all the money paid into the Fund, (*b*) without such refund. I have been induced to select that arrangement, because two days ago I received a letter from Miss Vincent, who is now in Switzerland, and who shows a very practical acquaintance with the question. (Letter read.)

Rates of Payment for Various Ages.

Now I come to the question of the rates of payment for the various ages under provision, and I have stuck at this point because I never was able to find anything like a consensus of opinion as to what nurses could pay. I took it, looking at the fact that nurses have everything provided for them except underclothing, that taking a nurse who received £25 a year, if she took £5 for amusements, £5 for pocket-money, and £10 for underclothing, she would have £5 left, which might go into the savings-bank. But £5, I have been told, is an impossible sum, that she has not got it to give, that she cannot spare it, and that if I prepared tables which necessitate the payment of £5 a year I should not get any number of nurses to join, because they cannot afford to pay that sum. Well, you must remember that after all it is not for our benefit but for your benefit that we propose to establish this Fund, and therefore, if you really want a Fund, and want it to help you, you must exercise some self-denial while you have health and strength to do it, that is, you must join early in life. You must remember, in considering this question of what you can pay, that the more you can pay the better it will be for you as you get older or permanently ill, or when you really want the charity.

What can a Nurse Pay?

Years ago it was my duty, as the representative of one of the great journals, to go into the tenement houses of a great city to ascertain the circumstances of the people, and see whether they were those of deep and dire distress. I and a gentleman who worked with me did not know how to form a basis as to what a man should pay for house room, which was a very important matter. Casting about, one of us read over the great Napoleon's Code, and he has laid it down that no one ought to devote more than one-eighth of his income for rent. What is that to do with this Fund, you may ask? Well, this much. The members who join this Fund, being engaged in public institutions and unmarried, will have no house rent to pay, and I thought, therefore, that they might possibly devote this eighth of their income to the Pension Fund, on this basis. I propose to treat you on this principle, and to see what an eighth of the ordinary income of a nurse and hospital official will produce by way of pension and sick-relief. For illustration I will take the two ages of twenty-five and thirty (although I could take any others) and give you an idea of what can be done. The figures are, as I have told you, round figures.

Minimum Payments and Results Expected.

First, a nurse who is about twenty-five years of age and receives £25 a year, on joining would pay to the Pension Fund £3 5s. per annum, or five shillings and sixpence a month, that is, one shilling and threepence per week. Very well, now I want to know what that will produce. If she determines that she will go for a pension at sixty years of age, it will produce £15 per annum, with the return of all her money at any time, or £18 without. If you get on, you will increase your pension, because if once you join you will get the full privileges, and you will be able to convert that £15 ultimately into something like £25 a year, or possibly into £30 a year. With bonus additions £30 would probably become in such a case £50 per annum as a retiring allowance for such a nurse at sixty years of age. That, then, is what you can do on the Napoleonic basis. Now to take the case of a sister, and assume that, having been a nurse, she is promoted at thirty. She receives £35 a year. Now an eighth equals £4 6s. 6d. That amount would provide a pension of £15 a year, or one of £21 a year without profits. A lady superintendent, thirty years of age, receives £80 a year. Or course, she may be much older than thirty, but I have taken thirty to keep the figures on one basis. An eighth of her income is £10, and that £10 a year would give her £38 a year if she receives profits, or £48 if she does not. I will now take a secretary. He receives at thirty years of age a salary of £250 a year. On the Napoleonic basis he would give £31 5s. to this Fund, which would entitle him to £114 per annum with, or £144 without profits. Now let us compare these rates with those which Guy's Hospital has been working since 1851. If you take Guy's Fund, a nurse commencing at £25 cannot participate till she is sixty-five. The Governors of Guy's Hospital are willing, and indeed consider it their duty and privilege, to contribute half of the annual payments which are necessary for a member to pay to enable them to get a pension of £20 or £30 per annum at sixty-five years of age. I commend that to the attentive and reflective consideration of every house and institution in this country. What committees can do apart from mere gifts of money is to adopt a uniform practice in the treatment of nurses, and live up to that. I find that there are many institutions which do not provide their nurses with washing, which is equal to about £4 a year. If every institution will provide you with washing you can pay your washing-money into the Fund, and you will be happy for ever more with a minimum pension of £50 per annum certain at sixty years of age. I know no organisation more calculated to produce a feeling of brotherhood and sisterhood than the association of workers within hospital walls, rightly understood. Those committees who look after their staff, and really devote themselves to the staff, will have their reward, and that reward will be meted out to them with no stinted hand.

Bonus Additions.

I should have liked to give you a number of instances of the application of bonuses. If Jane Smith is nursing Mr. Thomas, a barrister, through a dangerous illness, and Mr. Thomas, grateful for her care, instead of racking his brains to think what he can do in the way of presents, if he could know that a contribution of £25 could be paid to this Fund to the credit of Jane Smith, to provide for her when she was permanently invalided, that would be a grand result of a pension fund; it would give it an enormous impetus, and do enormous good by directing a huge volume of public sympathy towards nurses in general, and towards the individual nurse who has attended us through our illness. The question arises: Why is it necessary and desirable to establish a special fund instead of using the Post-office system? Why not go to the Post-office? The answer is: Because your circumstances are such that you cannot afford to pay premiums to provide an adequate pension unless the people conducting the Fund understand the difficulties under which you work, and will make public the fact, and will take the special bonuses, and make this fact known—that is one reason why; but I could give you several others.

Queen Victoria's Bounty.

One word with regard to a donation from the Queen's Fund. Why do we wish the Queen to give us a donation? It is that we can begin the Fund upon the widest basis by no other means, unless some one will send a cheque for £20,000. It is that the law will prevent us from beginning unless we have this sum. I do make bold to say that there is every reason to express the most earnest hope that, before the whole of that £75,000 is distributed, the most careful, thoughtful, and earnest attention will be given to this question of the Pension Fund. I cannot believe it possible that, when the prayer is properly presented in the right quarter, it will fail in its effect. When I tell you the law is as follows, it seems the best of all reasons why the Queen should give £20,000 to this Fund, and we should joyfully receive it. By the Friendly Societies Act, no friendly society may grant annuities to a greater amount than £50 per annum, and £20,000 must be invested in the names of trustees. It is there absolutely safe, and not going to be frittered away, and only the interest on that £20,000 could be devoted to pensions. Therefore we do not ask the Queen to give £20,000 to anyone, but to transfer £20,000 into Consols, in order to make it possible to found a Pension Fund. Now, if that point goes forward, as I hope it will go forward, I do not think we need fear that it will receive the most careful attention, and I hope a favourable response.

A Prospect Assured.

I have lived many years of my life in hospitals, and have seen very much of their work in the past. When I remember and know, as I do know, how trying the hospital life is, how it sometimes excites us to stir all our energies to their very depths to do our utmost for poor sufferers, and how sometimes we ourselves suffer by the diseases which we contract from those we live among and minister to—when I think of all these things (although I am now removed to another sphere of work), I do feel, looking at this large and representative gathering, that the same earnestness of purpose which animates me will animate you to-night. I only hope that this work, which has been a labour of love to me, will, by God's blessing and your help and self-denial, end in great results—nay, in magnificent results, because it will make it impossible for any of us ever again to see those who have ministered to the sick having no home or refuge but the workhouse to which they can go when illness or old age comes upon them.

